# Complete Genome Sequence of *Phenylobacterium* Strain NIBR 498073, Isolated from the Sediment of a Tidal Flat

**DOI:** 10.1128/mra.00080-23

**Published:** 2023-06-08

**Authors:** Gyungcheon Kim, Jin Young Park, Jin Nam Kim, Jaewoong Yu, Bongsang Kim

**Affiliations:** a Department of Food Science and Biotechnology, College of Life Science, Sejong University, Seoul, South Korea; b Biological Resources Research Department, National Institute of Biological Resources, Incheon, South Korea; c eGnome, Inc., Seoul, South Korea; d Department of Agricultural Biotechnology and Research Institute of Agriculture and Life Sciences, Seoul National University, Seoul, South Korea; University of Southern California

## Abstract

We report the complete genome sequence of *Phenylobacterium* sp. strain NIBR 498073. The sample was isolated from sediment from a tidal flat in Incheon, South Korea. The whole genome consists of one circular chromosome of 4,289,989 bp, and annotation using PGAP predicted 4,160 protein coding genes, 47 tRNAs, 6 rRNAs, and 3 noncoding RNAs.

## ANNOUNCEMENT

*Phenylobacterium* sp. strain NIBR 498073, which is Gram negative, flagellar, and rod shaped, was isolated from tidal flat sediments in Incheon, South Korea (1, 2). *Phenylobacterium* not only plays a role in maintaining the environment by participating in various metabolism processes in nutrient-poor environments but also has important roles in biodegradation (3–5). However, only 17 valid descriptions of *Phenylobacterium* species have been published so far (https://lpsn.dsmz.de/genus/phenylobacterium) (6).

A sediment sample taken from a depth of less than 5 cm at a tidal flat in Incheon, South Korea (37°39′02.0″N, 126°31′10.0″E), was diluted in 0.85% (wt/vol) saline, spread onto marine agar 2216 (BD), and then incubated at 25°C for 3 days. Cells from a single colony were collected in a 5-mL Eppendorf tube for DNA extraction. Genomic DNA was extracted using the RBC DNA extraction kit (Taiwan), and a Covaris g-TUBE device was used to shear the genomic DNA according to the manufacturer’s instructions. The DNA was sheared to a target size of 15 kb, purified using AMPure XP magnetic beads (Beckman Coulter, Inc.), and measured using a bioanalyzer (Agilent, USA). A library was constructed using the PacBio SMRTbell library preparation kit version 1.0 (Pacific Biosciences, Menlo Park, CA, USA). DNA damage repair and end repair were performed using the SMRTbell damage repair kit (Pacific Biosciences), and then the SMRTbell enzyme cleanup kit (Pacific Biosciences) was added to the library. The genome library size was measured using the Blue Pippin size selection system (Sage Science, Beverly, MA, USA) to ensure that all reads were over 15 kb, and the library was sequenced on the PacBio RS II platform.

To preprocess the raw sequence reads (*N*_50_, 9,998 bp), the SeqKit v2.3.0 was used to evaluate quality and filter reads less than 1,000 bp long (7). This step yielded 1,535,346 filtered sequence reads, with 13,303,322,204 bp. The bacterium formed a high-quality sequence, with a coverage of over 2,892×. As a result of assembly using the Flye version 2.9.1 pipeline (8), 1 complete circular genome sequence of 4,289,989 bp was constructed. After annotation based on the NCBI Prokaryotic Genome Annotation Pipeline (PGAP) version 6.5 (9), strain NIBR 498073 was found to contain 4,160 protein coding genes, 47 tRNAs, 6 rRNAs, and 3 noncoding RNAs (ncRNAs). All genomic sequence data are summarized in Table 1.

**TABLE 1:** Complete genome sequence assembly and annotation data for *Phenylobacterium* strain NIBR 498073

Parameter	Data
Assembly data (before/after filtering)	
Total no. of reads	1,567,148/1,535,346
Minimum read length (bp)	51/1,000
Avg read length (bp)	8,500/8,665
Maximum read length (bp)	197,345/197,345
*N*_50_ (bp)	9,998/10,004
Total size (bp)	13,320,171,249/13,303,322,204
Coverage (×)	2,895/2,892
Annotation results	
No. of genes	4,255
No. of CDSs^*a*^ (with protein)	4,160
No. of tRNAs	56
No. of rRNAs	6
No. of ncRNAs	3
Accession no.	
GenBank	GCA_027286305.1
BioProject	PRJNA913615
SRA	SRR22805297

a CDSs, coding DNA sequences.

The 16S rRNA sequence was matched using BLASTn with the NCBI bacteria and archaea 16S rRNA database (10). The most similar bacterial strain was Phenylobacterium parvum strain HYN0004 (GenBank accession number CP029479.1), with an identity of 96.89%, followed by Caulobacter mirabilis strain FWC 38 (CP024201.1; identity, 96.62%), Phenylobacterium zucineum strain HLK1 (CP000747.1; identity, 75.82%), and Phenylobacterium cathodiphilus strain S-12 (CP027668.1; identity, 70.46%). In addition, the average nucleotide identity (ANI) was calculated using Pyani (11). The closest match was Phenylobacterium haematophilum strain DSM 21793 (GCF_014196295.1), with an ANI value of 88.99 ± 3.96%. Default parameters were used for all software unless otherwise specified.

The complete genome of strain NIBR 498073 will provide insight into isolating novel *Phenylobacterium* species and contribute to a deeper understanding of the biological properties of bacteria.

### Data availability.

The accession numbers are listed in Table 1. The assembled genome was deposited at GenBank under the accession number CP114599 and the BioProject accession number PRJNA913615. The raw sequence reads have been submitted to the Sequence Read Archive (SRA) under the accession number SRR22805297.
